# Adherence to the Mediterranean Diet and Cardiovascular Risk Factors among the Lebanese Population: A Nationwide Cross-Sectional Post Hoc Study

**DOI:** 10.3390/nu16152426

**Published:** 2024-07-26

**Authors:** Rony M. Zeenny, Chadia Haddad, Aline Hajj, Rouba K. Zeidan, Pascale Salameh, Jean Ferrières

**Affiliations:** 1 Department of Mathématiques Informatique et Télécommunications, Université Toulouse III, Paul Sabatier, INSERM, UMR 1295, F-31000 Toulouse, France; 2 INSPECT-LB (Institut National de Santé Publique, d’Épidémiologie Clinique et de Toxicologie-Liban), Beirut 1103, Lebanon; chadia_9@hotmail.com (C.H.); aline.hajj@hotmail.com (A.H.); roubakaren@gmail.com (R.K.Z.); pascalesalameh1@hotmail.com (P.S.); 3 Department of Pharmacy, American University of Beirut Medical Center, Beirut 1107, Lebanon; 4 Gilbert and Rose-Marie Chagoury School of Medicine, Lebanese American University, Byblos 4504, Lebanon; 5 School of Health Sciences, Modern University of Business and Science, Beirut 7501, Lebanon; 6 Research Department, Psychiatric Hospital of the Cross, Jal El Dib 1525, Lebanon; 7 Faculté de Pharmacie, Université Laval, Québec, QC G1V 0A6, Canada; 8 Oncology Division, CHU de Québec-Université Laval Research Center, Québec, QC G1R 3S3, Canada; 9 Sharjah Institute of Medical Research, University of Sharjah, Sharjah 27272, United Arab Emirates; 10 Faculty of Pharmacy, Lebanese University, Hadat 1103, Lebanon; 11 Department of Primary Care and Population Health, University of Nicosia Medical School, 2417 Nicosia, Cyprus; 12 Department of Cardiology and INSERM UMR 1295, Rangueil University Hospital, F-31059 Toulouse, France; jean.ferrieres@univ-tlse3.fr

**Keywords:** adherence, cardiovascular diseases, dietary patterns, healthy diet, Lebanon, lifestyles, Mediterranean diet, non-communicable diseases, nutrition, risk factors

## Abstract

Objective: This study aims to identify the association between adherence to healthy eating, using the Lebanese Mediterranean Diet Scale (LMDS), and cardiovascular risk factors in the Lebanese population. Materials and Methods: A cross-sectional study using a multistage cluster sample was conducted in Lebanon. Sociodemographic characteristics were collected through structured interviews and self-administered questionnaires. The LMDS assessed dietary habits. The associations between diabetes, dyslipidemia, and cardiovascular disease were investigated using stratification analysis. Results: The study included 2048 people (mean age: 41.54 ± 17.09 years). Higher adherence to the Mediterranean diet was associated with older age (Beta = 0.175, *p* < 0.001), being female (Beta = 0.085, *p* = 0.001), being married (Beta = 0.054, *p* = 0.047), participating in regular physical activity (Beta = 0.142, *p* < 0.001), and having cardiovascular disease (Beta = 0.115, *p* < 0.001) and diabetes (Beta = 0.055, *p* = 0.043). Adherence was, however, negatively associated with being a smoker (Beta = −0.083, *p* = 0.002), a previous smoker (Beta = −0.059, *p* = 0.026), and having higher distress levels (Beta = −0.079, *p* = 0.002). Stratification analysis by diabetes, dyslipidemia, and cardiovascular disease (CVD) consistently demonstrated these associations. Conclusions: These findings suggest that demographic and health factors influence the Lebanese population’s adherence to the Mediterranean diet. Older age, female gender, married status, physical activity, CVD, and diabetes were all found to be associated with adherence to the Mediterranean diet in the Lebanese population. In contrast, smoking and distress were inversely associated with it.

## 1. Introduction

Cardiovascular disease (CVD) is a significant global public health concern [1], being the leading cause of nutrition-related mortality (10 million deaths) and disability-adjusted life years (DALYs) (207 million) [2,3,4]. Several risk factors contribute to the development and progression of CVD, with diet emerging as a crucial modifiable risk factor [5].

The Mediterranean diet (MedDiet), introduced by Ancel Keys in the 1950s as a part of his landmark Seven Countries Study [6,7], has been highlighted for its association with reduced incidence of CVD among specific communities [6,7]. This recognition led to extensive research exploring its beneficial effects. In 2010, the United Nations Educational, Scientific and Cultural Organization (UNESCO) recognized the MedDiet as an Intangible Cultural Heritage of Humanity [8]. Moreover, the World Health Organization Ministerial Conference recognized in 2013 that “a healthy diet can contribute to achieving the global targets on non-communicable diseases (NCD), including achieving a 25% relative reduction in NCD’s premature mortality by 2025” [9]. Along the same line, the 2030 United Nations (UN) Agenda’s third Sustainable Development Goal (SDG) aims to reduce premature deaths from non-communicable diseases by one third by 2030, alongside promoting mental health and wellbeing (SDG Target 3.4) [10].

The MedDiet derives its nutritional principles from the traditional eating habits of countries bordering the Mediterranean Sea [11]. However, it manifests in various forms within and across those countries. Variations are influenced by local food availability, cultural preferences, and socio-economic conditions [12]. The MedDiet emphasizes a high consumption of plant-based foods, like cereals and whole grains, fresh fruit and vegetables, legumes, nuts, and olive oil. It also promotes low-to-moderate intake of fish and poultry, limited consumption of red meat, eggs, and processed foods, and moderate wine consumption, often with meals [4,12]. This dietary pattern, rich in plant-based foods, offers numerous health benefits attributed to its antioxidant and fiber-rich content and abundance of polyunsaturated fatty acids (PUFA) and monounsaturated fatty acids (MUFA) from sources like fish, nuts, and olive oil. Conversely, it has lower levels of saturated and trans-fatty acids in meats and sweets [13].

Today, the MedDiet stands as one of the most extensively studied eating patterns worldwide [7,14]. Meta-analyses provide robust evidence of a significant inverse association between adherence to the MedDiet and various health outcomes, including all-cause mortality, CVD [13,14,15], and several cardiometabolic risk factors, such as metabolic syndrome [16], type 2 diabetes (T2DM) [17], and obesity [18].

Previous meta-analyses and prospective studies, pooling fatal and non-fatal CVD events, have revealed that even a modest increase in MedDiet adherence score, typically by two points, allows a notable 10% reduction in CVD risk. However, specific analyses related to outcomes like coronary heart disease (CHD), myocardial infarction (MI), and stroke are relatively scarce [14]. Furthermore, true meta-analyses only include research that used a specific dietary index and only observational studies [14].

For secondary CVD prevention, the Lyon Diet Heart Study, a groundbreaking randomized clinical trial [19], revealed compelling findings. This study involved approximately 600 participants and demonstrated that adopting a MedDiet pattern rich in alpha-linoleic acid significantly reduced the risk of recurrent events compared to a prudent western-type diet following a first MI. Specifically, participants adhering to the Mediterranean diet experienced a remarkable 72% reduction in the incidence of cardiac death and non-fatal MI over up to four years post-MI [19].

After the Lyon Diet Heart Study, numerous prospective studies have supported its findings, providing substantial evidence supporting the protective effects of the Mediterranean diet against recurrent cardiac events [20]. Additionally, research indicates that adherence to the MedDiet is associated with improved survival following a diagnosis of CVD [21]. In another secondary prevention study conducted in India [22], the MedDiet reduced cardiac endpoints, sudden deaths, and non-fatal MI after two years of follow-up, although its reliability is questionable. Similar findings were reported from the GISSI-Prevenzione clinical trial in Italy, indicating that Mediterranean-type diets reduced mortality post-MI, particularly with increased consumption of fish, fruit, vegetables, and olive oil [23]. More research further supported these outcomes, revealing a decreased risk of mortality risk among MI patients adhering to the MedDiet [24].

Assessing dietary trends is challenging due to the inability to observe and measure them directly. Consequently, several techniques have been developed to define these patterns based on dietary data [15].

Several indexes or scores have been developed and proposed to evaluate adherence to this dietary pattern, with their effectiveness in predicting health outcomes in numerous longitudinal studies [25]. The Lebanese Mediterranean Diet Score (LMDS) [26], calculated by Naja et al., offers a robust and straightforward measure to assess adherence to the Middle Eastern variant of the MedDiet. Notably, it was significantly correlated with all European MedDiet indexes, showing the closest resemblance to the Italian MedDiet [26]. Aside from fruit, vegetables, and olive oil, the LMDS incorporates burghul, dried fruit, and dairy products, emblematic of traditional Lebanese cuisine and other countries in the Eastern Mediterranean nations.

To the best of our knowledge, comprehensive studies analyzing the correlation between adherence to healthy eating and cardiovascular (CV) risk factors, particularly concerning secondary prevention of CVD, remain scarce in the Lebanese context. In light of this research gap, this study aims to elucidate the relationship between adherence to healthy eating, as measured by the LMDS, and CVD and associated risk factors within the Lebanese population.

## 2. Materials and Methods

### 2.1. Design and Population

The study’s methodology has been previously described [27,28]. Data were collected through a cross-sectional survey conducted between September 2013 and October 2014, using a multistage cluster sampling technique across Lebanon [28]. Out of Lebanon’s 2789 circumscriptions (villages or settlements regarded as clusters), 100 were randomly selected using an automatic random number generator.

The Lebanese Central Administration of Statistics provided a comprehensive list of circumscriptions. The selection of inhabitants aged ≥18 years old was conducted using a software program to assure randomness, using a list of residents provided by the local administrations. Participants underwent a face-to-face interview following verbal and written consent. Individuals with recognized mental illnesses or learning difficulties were excluded. Given the observational nature of this study, the institutional review board of the Lebanese University waived the need for ethical approval.

### 2.2. Sample Size

Initial sample size calculations were performed to determine the prevalence of CVD and its associated risk factors. Epi Info^®^ from the Centers for Disease Control in Atlanta, GA, USA was used for this purpose [29] accessible at http://www.cdc.gov/epiinfo/, last accessed on 4 May 2024). To estimate the prevalence of CVD in an adult population aged 40 years and older in Lebanon, a sample size of at least 1200 was deemed necessary. We used a baseline reference [30] citing the prevalence of arterial hypertension (41.3% among those 50 years of age and above), a ±4% margin of difference, a 95% confidence interval (CI), and the two-stage sample method.

### 2.3. Data Collection

A standardized questionnaire was used to collect the following self-reported information: sociodemographic characteristics such as age, gender, marital status, educational level, region of residence, occupation, and socio-economic status. Participants also provided information on their medical history, including heart diseases, other medical conditions, and medication used for glucose control and lipid-lowering. Social behaviors like smoking and physical activity were also recorded. Socio-economic status was categorized into four groups based on the household’s total monthly income in United States Dollars (USD): low (≤USD 400, the equivalent of the minimum wage in Lebanon), intermediate low (USD 400–1000), intermediate high (USD 1000–2000), and high (>USD 2000).

Participants were asked if they had hypertriglyceridemia or hypercholesterolemia, with confirmation of responses sought through laboratory test findings or prescription drug records. Family history of premature CVD was considered if a first-degree relative had CVD before age 55 for men and 65 for women [31,32].

Psychological distress was assessed using the Beirut Distress Scale (BDS-22), specifically designed and validated among Lebanese individuals. The BDS-22 employed a four-point (0–3) Likert-type response format across the 22 items, measuring psychological discomfort on a scale ranging from 0 to 66 (highest) [33].

Dietary practices were evaluated using the Lebanese MedDiet Score (LMDS), a 16-item questionnaire tailored to assess adherence to a MedDiet within the Lebanese context. It covers the various food categories that Lebanese usually consume. The score, ranging from 0 to 64 (maximal adherence), measures adherence to the MedDiet [26,34]. The study did not assess any alcohol intake as its consumption would be underreported due to a religious ban.

### 2.4. Measurements

Trained medical students conducted anthropometric measurements, including weight (in kg), height (in m), and waist circumference (WC) (in cm). Body mass index was determined by dividing weight in kilos by height in square meters. Body Mass Index (BMI) categories were defined as follows: normal weight (BMI < 25 kg/m^2^), overweight (25 kg/m^2^ ≤ BMI < 30 kg/m^2^), and obese (BMI ≥ 30 kg/m^2^). Abdominal obesity was defined using WC cutoff standards of 92.5 cm for women and 93.5 cm for males [35]. Systolic and diastolic blood pressure measurements were taken twice using the Omron^®^ M6 Comfort (Omron^®^, Kyoto, Japan), electronic automatic equipment following standardized procedures [36]. Random capillary blood glucose (RCBG) testing was performed using the Accu-Check^®^ Performa (Roche Diagnostics GmbH, Mannheim, Germany).

### 2.5. Definitions

Definitions of CVD: Participants disclosing a prior MI, percutaneous coronary intervention, or coronary artery bypass graft were considered eligible. The Rosa Angina Questionnaire’s [37] definition of “definite angina” was used to describe angina. Prevalence rates of MI, angina pectoris, percutaneous coronary intervention, and coronary artery bypass graft were compiled to ascertain the lifetime prevalence of coronary disease. Additionally, a positive response to the question, “Has the doctor ever told you that you had a stroke or a mini-stroke?” was also used to determine the presence of cerebrovascular disease. CVD, including coronary or cerebrovascular diseases, was the primary dependent variable.

Risk Factors Definitions: Hypertension was defined as a self-reported history of hypertension or repeated readings of ≥140 mmHg for systolic blood pressure or ≥90 mmHg for diastolic blood pressure [31,32,38]. Diabetes was defined as having an RCBG of 200 mg/dL or more or self-reported use of medication to regulate blood sugar. Dyslipidemia was defined as self-reported previously diagnosed hyperlipidemia with or without current hypolipemic treatment. Participants providing their latest blood tests showing hyperlipidemia were also included in this group [39].

“Current smokers” refers to individuals who had smoked tobacco within the past 12 months, including people who have recently given up (less than one year). Former smokers had quit more than a year prior. A history of heart disease was defined as any self-reported myocardial infarction, stenting, angioplasty, or coronary artery bypass graft.

Participants were considered physically active if they regularly engaged in vigorous-intensity physical exercise for at least 75 min per day or moderate-intensity physical activity for at least 150 min per week [40].

### 2.6. Statistical Analysis

Two independent observers double-checked the accuracy of the questionnaire data before statistical analysis, and an additional audit was carried out on a random sample of 5% of the questionnaires. The distribution of adults in Lebanon’s regions was theoretically calculated using population data from the Central Administration of Statistics (CAS) and the Lebanese Ministry of Social Affairs [41], accounting for sex, age, and dwelling region.

The sample was then weighted to fit this distribution and reflect the Lebanese population’s characteristics. Without changing the sample size overall, participants who were underrepresented in the sample had their weights increased, while those who were overrepresented had their weights decreased. Statistical Package for the Social Sciences (SPSS) software version 25 was used to analyze the data. Concerning categorical variables, counts and percentages, as well as means and standard deviations, were used in a descriptive analysis.

When calculating the LMDS total score, random missing values were encountered, and a correction analysis of those values was conducted (replacement by the mean). No difference in the variance was reported before and after the missing value replacement. The sample was normally distributed, as checked by visual inspection of the histogram of the LMDS scale and verified by the normality line of the regression plot and scatter plot of the residual. After checking the normality of the variables, the independent-sample *t*-test was used to compare the means between two groups, and the analysis of variance (ANOVA) test was applied to compare three or more means.

The linear regression models included all variables with a *p*-value < 0.1 in the bivariate analysis to avoid potential confounders. A multivariate analysis (multiple linear regression models) was carried out using the Enter method, taking the LMDS scale as the dependent variable. In addition, three stratification analyses were performed according to diabetes, dyslipidemia, and CVD, taking the LMDS scale as the dependent variable. A *p*-value < 0.05 was considered significant.

## 3. Results

### 3.1. Sample Description

The sociodemographic characteristics of the participants are presented in Table 1. Among them, 51.5% were female, and 54.4% were aged between 18 and 39 years. The majority (54.6%) were married, had a school-level education (54.6%), and were employed (61.3%). A significant proportion reported a low individual monthly income (54.2%) and resided primarily in Mount Lebanon and Beirut areas (53.8%). The mean age of the participants was 41.54 ± 17.09 years, with a mean individual monthly income of 391.19 ± 499.46 USD. Additionally, 7.66% of the sample were on hypoglycemic medications, and 8.59% used lipid-lowering agents.

### 3.2. Bivariate Analysis: LMDS Scale

Table 2 shows the bivariate analysis, taking the LMDS scale as the dependent variable. The results showed that females exhibited higher adherence to the Mediterranean diet, reflected by a higher LMDS score compared to males (*M*_male_ = 30.17 vs. *M*_female_ = 30.90, *p* = 0.002). Additionally, a higher mean LMDS scale was found among married participants compared to single individuals (*M*_single_ = 29.85 vs. *M*_married_ = 31.02, *p* < 0.001) and among those over 60 years compared to other age groups. Nonsmokers with regular physical activity showed a higher mean LMDS (*M*_yes_ = 31.52 vs. *M*_No_ = 30.04, *p* < 0.001), alongside the unemployed participants (*M*_unemployed_ = 30.86 vs. *M*_employed_ = 30.32, *p* = 0.023). Furthermore, participants with CVD (*M*_yes_ = 31.70 vs. *M*_No_ = 30.15, *p* < 0.001), hypertension (*M*_yes_ = 31.19 vs. *M*_No_ = 30.20, *p* < 0.001), diabetes (*M*_yes_ = 31.88 vs. *M*_no_ = 30.20, *p* < 0.001) and dyslipidemia (*M*_yes_ = 31.58 vs. *M*_No_ = 30.25, *p* < 0.001) exhibited higher mean LMDS scores. Age also demonstrated a significant association with adherence to the MedDiet (higher LMDS scale) (r = 0.217, *p* < 0.001). The means of adherence to LMDS food elements are provided as Supplementary Material in Table S1.

### 3.3. Multivariable Analysis

A linear regression analysis was performed, taking the LMDS as the dependent variable. The results showed that higher age (Beta = 0.047), being a female (Beta = 0.794), being married (Beta = 0.510), doing regular physical activity (Beta = 1.400), and having CVD (Beta = 1.269) and diabetes (Beta = 0.679) were significantly associated with higher adherence to the MedDiet (higher LMDS scale). However, being a smoker (current, Beta = −0.777 or previous smoker, Beta = −1.003) and having higher psychological distress levels (Beta = −0.032) were significantly associated with lower adherence to the MedDiet (Table 3). 

The results of the stratification analysis according to diabetes, dyslipidemia, and CVD subgroups are presented in Table 4, Table 5 and Table 6, respectively. When examining the analysis stratified by diabetes, results showed that regular physical activity was significantly associated with a higher LMDS scale in both diabetic and non-diabetic groups. Among participants without diabetes, the analysis revealed that higher age (Beta = 0.05), female gender (Beta = 0.91), and the presence of CVD (Beta = 1.31) were significantly associated with increased adherence to the MedDiet (higher LMDS scale). Conversely, being a smoker (Beta = −0.95), having hypertension (Beta = −0.68), and experiencing higher levels of distress (Beta = −0.03) were significantly associated with decreased adherence to the MedDiet (Table 4).

The results of the stratification analysis according to dyslipidemia are displayed in Table 5. Results showed that higher age and regular physical activity were significantly associated with higher LMDS scales in both dyslipidemia and non-dyslipidemia groups. Among participants without dyslipidemia, the analysis showed that being female (Beta = 1.03) and having CVD (Beta = 1.73) were significantly associated with increased adherence to the MedDiet (higher LMDS scale). However, being a current smoker (Beta = −0.84) or a previous smoker (Beta = −1.16) and exhibiting higher levels of distress (Beta = −0.04) were significantly associated with lower adherence to the MedDiet. Among the participants in the dyslipidemia subgroup, the results showed that being married (Beta= 2.53) was significantly associated with higher adherence to the Mediterranean diet (higher LMDS scale).

Finally, the analysis of CVD subgroups found that higher distress levels were significantly associated with a lower LMDS scale in both groups. When considering participants without CVD, higher age (Beta = 0.06), being female (Beta = 1.21), and having regular physical activity (Beta = 1.51) were significantly associated with increased adherence to the MedDiet (higher LMDS scale). However, being a smoker (Beta = −0.76) and experiencing higher distress levels (Beta = −0.02) were significantly associated with lower adherence to the Mediterranean diet.

In contrast, among participants with CVD, only diabetes (Beta = 1.49) was significantly associated with higher adherence to the Mediterranean diet (higher LMDS scale) (Table 6).

## 4. Discussion

This study highlights that individuals with pre-existing conditions demonstrate better adherence to the MedDiet, whereas those smoking and exhibiting higher levels of distress had lower adherence scores. Our findings suggest that people with CVD and dyslipidemia had slightly higher adherence to the MedDiet compared to those without. These results contrast other cross-sectional studies, showing that MedDiet adherence is lower among CVD patients [42,43,44,45]. The MedDiet’s benefits stem from its high content of nutraceuticals and an abundance of bioactive compounds and essential micronutrients, fibers, and antioxidants from plant foods, particularly polyphenols and vitamins in fresh vegetables and fruit or omega-3 polyunsaturated fatty acids (PUFAs) in fish and seeds [46]. It also includes complex carbohydrates and monounsaturated fats while restricting the intake of animal fats and sweets., This diet favorably impacts blood pressure, body weight, glycemic control, vascular inflammation, and arteriosclerosis, suggesting its preventative mechanism [47]. The MedDiet may enhance glucose metabolism, insulin sensitivity, lipid profile, and CVD risk management, ultimately being protective against CVD. One possible explanation for our findings is that people improve their diet quality in response to receiving a CVD, Diabetes, and/or Dyslipidemia diagnosis [19,20,21]. A review by Low et al. showed that nutritional counseling enhances cardiometabolic health in middle-aged and older adults [48]. This finding supports the idea that adherence to the MedDiet may improve with aging [48]. Additionally, a recent meta-analysis by Laffond et al. [49] indicates that both the general population and individuals with prior CVD may experience positive effects from following the Mediterranean diet.

Furthermore, this study reveals moderate overall adherence to the MedDiet among participants. This aligns with previous research among 3384 Lebanese university students, indicating a similar moderate adherence to the MedDiet [34] and two other Lebanese studies reporting moderate to high adherence to the MedDiet among the general population [50,51]. Also, a survey done among 367 Lebanese and Syrian adults found that only 47.42% of participants reported moderate to good adherence (≥9 points over 14) to MedDiet [52]. These discrepancies in the literature could be explained by variations in study design, methodology, sampling characteristics, and the approach to evaluating the MedDiet. Additionally, the observed moderate adherence in our study may be attributed to older individuals gravitating toward conventional eating patterns compared to younger ones, who may be exposed to novel and “trendy” food products [53].

Moreover, we found higher adherence among women than men, aligning with previous research [35], which shows that females exhibit higher dietary adherence than males. Women tend to prioritize healthier food choices and maintain appropriate eating habits to preserve good physical shape [54].

Furthermore, unhealthy lifestyle choices, such as smoking, were expectedly associated with poor adherence to the MedDiet. A study done among 193 university students from Cyprus has found that smokers exhibited lower adherence to MedDiet compared to nonsmokers [55]. Similarly, another study involving 841 women showed that individuals with high adherence to the MedDiet were less likely to smoke tobacco [56]. It is well-established that smokers often adopt unhealthy dietary habits characterized by higher intakes of total calories, saturated fats, cholesterol, and alcohol, along with lower intakes of antioxidant vitamins and dietary fiber, a worse diet than nonsmokers [57,58,59,60,61].

Our study revealed a positive correlation with a higher adherence to the MedDiet regarding physical activity. This aligns with previous research conducted among 326 Lebanese adults, where physical activity was strongly associated with higher MedDiet adherence levels [53]. Similarly, a study has found better adherence to the Lebanese MedDiet among 3384 Lebanese students who engaged in higher levels of physical activity and smoked less tobacco [34]. Another Lebanese study among 525 university students found that nonsmokers tended to score more favorably on the MedDiet and achieved higher mean scores compared to smokers [62]. Thus, individuals with a favorable lifestyle tend to associate healthy behaviors: Adopting a healthy lifestyle with regular physical activity, reduced smoking, and the consumption of a balanced diet is crucial for achieving optimal health-related body composition and overall wellbeing [63]. Also, managing high blood pressure and its consequences often involves a combination of regular physical exercise and adherence to a healthy dietary regimen among adults [64,65].

When stratifying by pre-existing medical conditions, our results showed a significant association between physical activity and higher adherence to the MedDiet among individuals with dyslipidemia and diabetes. Patients with established metabolic conditions are thus starting to apply healthier lifestyle measures: several research studies [66,67] have suggested a strong relationship between physical activity level and MedDiet adherence that, in turn, has numerous advantages to health, including a decreased risk of acquiring and worsening of diabetes, obesity, hypertension, in addition to depression and cognitive problems [68,69,70,71,72]. Our results are similar to a recent study involving 500 participants with diabetes, where adherence to the MedDiet was reported as 5.4% for low adherence, 77.2% for moderate adherence, and 17.4% for high adherence [73]. For individuals managing diabetes and dyslipidemia, adopting proper nutrition habits and healthy eating patterns is crucial to prevent chronic complications associated with the disease [74,75,76].

Also, the current study results revealed that pre-existing diabetes was associated with higher adherence to MedDiet among participants with cardiovascular disease, further suggesting a reverse causality pattern, where participants with comorbidities better adhered to MedDiet. Numerous studies have linked the MedDiet with a reduced incidence of cardiovascular events and mortality [77,78,79]. At the same time, patients with diabetes who adhere to a MedDiet may experience reductions in all-cause and CV-related mortality [78]. Therefore, well-controlled glycemic levels with low-carbohydrate diets and the MedDiet can serve as secondary prevention for CV events in clinical practice [44].

The current study also showed that psychological distress was associated with lower adherence to the MedDiet among patients with CVD, as shown by other researchers [80,81]. Although acute stress exposure may shut and suppress appetite by releasing the corticotropin hormone, Psychosocial and emotional factors like loneliness, stress, or depression have been directly linked to alterations in eating patterns, leading to a gradual decline in adherence to the MedDiet [82,83,84] thus increasing susceptibility to consuming calories-rich, high-transfat, high salt, and high-sugar foods, often associated with obesity [80,85]. Along the same lines, a recent cross-sectional analysis by Lisa et al. showed that adherence to MedDiet is inversely associated with the severity of anxiety and stress symptoms but not depression [86].

### 4.1. Practical Implications

Given the significant CV benefits associated with MedDiet, the public health sector must implement initiatives and programs to promote adherence to this dietary pattern as primary prevention [13,14,15] and not only as potentially secondary prevention [19,20,21], similar to the findings of this study. Healthcare professionals should encourage healthy eating and lifestyle choices at both individual and community levels, which can lead to improved health outcomes, enhanced quality of life, academic performance, physical fitness, and mental wellbeing while also serving to prevent obesity, diabetes, cardiovascular diseases, cancer, and mortality [87].

### 4.2. Limitations and Strengths

This study has several limitations that may impact the generalizability of our findings. First, residual or unmeasured confounding cannot be completely ruled out due to the observational nature of our study. Second, the cross-sectional design, while useful for establishing associations, does not prove causality and may need further longitudinal and interventional studies to be confirmed. Third, our study participants were predominantly white Caucasians; thus, our findings may not apply to other ethnic groups. Moreover, reliance on self-reported data for many variables and covariates, including nutritional information, introduces the possibility of recall bias. Despite efforts to mitigate this, inherent measurement errors in dietary assessments remain unavoidable. Nevertheless, the substantial sample size likely mitigates the impact of random misclassification. Apart from the large sample size, the key strengths of this study are the standardized data collection methods, the use of a validated and systematic questionnaire for dietary assessment, and recruitment across different genders and geographical regions in Lebanon. Additionally, our study sample is also representative of the general adult population. Nevertheless, a longitudinal study with multiple diet measures is warranted to confirm the suggested findings, including the clinical significance of the results.

## 5. Conclusions

The study examined the correlation between adherence to the Lebanese MedDiet Score and CV risk factors in the Lebanese population. Results showed that older age, female gender, married status, regular physical activity, and the presence of CVD or diabetes were all associated with higher adherence to the MedDiet, which may suggest that diet improves after CVD or metabolic disease has occurred.

Adherence was also negatively associated with smoking status, history of smoking, and elevated distress levels. Stratification analysis revealed that these associations remained consistent across subgroups defined by the presence or absence of diabetes, dyslipidemia, or CVD. This suggests that demographic factors and health conditions play a significant role in influencing MedDiet adherence in Lebanon. Promoting a healthy diet and other behaviors like physical activity and stress management in primary prevention programs could improve adherence and reduce disease occurrence. Longitudinal studies are needed to explore additional factors affecting dietary adherence over time.

## Figures and Tables

**Table 1:** Sociodemographic Characteristics of Participants (N = 2048).

	Total
**Gender**	
Male	994 (48.5%)
Females	1054 (51.5%)
**Age (Class, years)**	
18–39	1116 (54.5%)
40–59	576 (28.1%)
60+	356 (17.4%)
**Marital Status**	
Single/Divorced/Widowed	924 (45.4%)
Married	1110 (54.6%)
**Education Level**	
Complementary	676 (33.2%)
Secondary	435 (21.4%)
University	721 (35.4%)
Postgraduate	203 (10.0%)
**Geographical Residence Distribution**	
Beirut	284 (14.0%)
Mount Lebanon	808 (39.8%)
Bekaa	245 (12.1%)
North Lebanon	396 (19.5%)
South Lebanon	296 (14.6%)
**Residence Area**	
Urban	512 (25.9%)
Rural	1001 (50.7%)
In between	460 (23.3%)
**Occupation**	
Unemployed + Retired	792 (38.7%)
Employed	1253 (61.3%)
**Categories of Individual Monthly Income**	
Low	550 (26.8%)
Lower intermediate	512 (27.4%)
Higher intermediate	458 (24.5%)
High	347 (18.6%)
**Age (years)**	
Mean ± SD	41.54 ± 17.09
**Individual Monthly Income (USD)**	
Mean ± SD	391.19 ± 499.46

**Abbreviations:** SD: Standard deviation; USD: United States Dollar.

**Table 2:** Bivariate analysis taking the LMDS total scale as the dependent variable.

	LMDS Scale Mean ± SD	*p*-Value
**Gender**		**0.002**
Male	30.17 ± 4.84	
Females	30.90 ± 4.59	
**Age, Class, years**		**<0.001**
18–39	29.43 ± 4.61	
40–59	31.35 ± 4.67	
60+	31.91 ± 4.49	
**Marital Status**		**<0.001**
Single/Divorced/Widowed	29.85 ± 4.96	
Married	31.02 ± 4.48	
**Education Level**		0.973
School	30.53 ± 4.81	
University	30.52 ± 4.66	
**Smoking status**		**0.001**
No	31.00 ± 4.35	
Yes	30.12 ± 4.97	
Previous smoker	30.79 ± 4.79	
**Regular physical activity**		**<0.001**
No	30.04 ± 4.64	
Yes	31.52 ± 4.77	
**Occupation**		**0.023**
Unemployed + Retired	30.86 ± 4.73	
Employed	30.32 ± 4.72	
**Categories of Individual monthly income**		0.431
Low	30.65 ± 4.57	
Lower intermediate	30.80 ± 4.67	
Higher intermediate	30.42 ± 4.44	
High	30.26 ± 5.06	
**Family history of CVD**		0.112
Yes	30.76 ± 4.73	
No	30.38 ± 4.73	
**CVD**		**<0.001**
Yes	31.70 ± 4.86	
No	30.15 ± 4.63	
**Hypertension**		**<0.001**
Yes	31.19 ± 4.80	
No	30.20 ± 4.67	
**Diabetes**		**<0.001**
Yes	31.88 ± 4.98	
No	30.20 ± 4.61	
**Dyslipidemia**		**<0.001**
Yes	31.58 ± 4.72	
No	30.25 ± 4.70	
	**Correlation coefficient**	
**Age (years)**	0.217	<0.001
**BMI**	0.038	0.127
**BDS-22**	−0.043	0.090

**Abbreviations:** BDS-22: Beirut distress scale; BMI: Body mass index; CVD: Cardiovascular diseases; LMDS: Lebanese Mediterranean Diet Score; SD: Standard deviation; ***p*-values in bold:** Statistically significant values.

**Table 3:** Multivariable analysis in the full sample.

Linear Regression Takes the LMDS Scale as the Dependent Variable					
	Unstandardized Beta	Standardized Beta	*p*-Value	Confidence Interval	
				Lower Bound	Upper Bound
Gender (Female vs. Male *)	0.794	0.085	**0.001**	0.307	1.282
Age	0.047	0.175	**<0.001**	0.030	0.064
Marital status (Married vs. single *)	0.510	0.054	**0.047**	0.008	1.012
Smoking (yes vs. No *)	−0.777	−0.083	**0.002**	−1.268	−0.285
Smoking (previous vs. No *)	−1.003	−0.059	**0.026**	−1.888	−0.118
Regular physical activity (yes vs. no *)	1.400	0.142	**<0.001**	0.921	1.879
Work status (Employed vs. unemployed *)	0.312	0.033	0.228	−0.196	0.821
CVD (yes vs. no *)	1.269	0.115	**<0.001**	0.700	1.839
Hypertension (yes vs. no *)	−0.330	−0.033	0.257	−0.900	0.240
Diabetes (yes vs. no *)	0.679	0.055	**0.043**	0.023	1.335
Dyslipidemia (yes vs. no *)	0.281	0.024	0.379	−0.346	0.909
BDS-22	−0.032	−0.079	**0.002**	−0.052	−0.011

* Reference group. Abbreviations: BDS-22: Beirut distress scale; CVD: Cardiovascular diseases; LMDS: Lebanese Mediterranean Diet Score; Variables entered in the model: Age; BDS-22; CVD; Diabetes; Dyslipidemia; Dyslipidemia; Gender; Hypertension; Marital Status; Occupation; Regular physical activity; Smoking status. ***p*-values in bold:** Statistically significant values.

**Table 4:** Multivariable analysis stratified according to diabetes.

Linear Regression Analysis Taking the LMDS Scale as the Dependent Variable				
	No Diabetes Subgroup		Diabetes Subgroup	
	UB (95% CI)	*p*-Value	UB (95% CI)	*p*-Value
Gender (Female vs. Male *)	0.91 (0.39; 1.43)	**0.001**	0.03 (−1.31; 1.38)	0.958
Age	0.05 (0.03; 0.07)	** <0.001 **	0.02 (−0.02; 0.06)	0.345
Marital status (Married vs. single *)	0.31 (−0.23; 0.85)	0.261	0.84 (−0.67; 2.36)	0.273
Smoking (yes vs. No *)	−0.95 (−1.48; −0.43)	** <0.001 **	0.14 (−1.18; 1.48)	0.828
Smoking (previous vs. No *)	−0.91 (−1.90; 0.08)	0.072	−0.96 (−3.00; 1.06)	0.349
Regular physical activity (yes vs. no *)	1.25 (0.73; 1.76)	** <0.001 **	1.80 (0.51; 3.10)	**0.006**
Work status (Employed vs. unemployed *)	0.27 (−0.27; 0.81)	0.331	0.11 (−1.33; 1.55)	0.881
CVD (yes vs. no *)	1.31 (0.68; 1.95)	** <0.001 **	1.00 (−0.34; 2.33)	0.143
Hypertension (yes vs. no *)	−0.68 (−1.30; −0.05)	** 0.032 **	0.98 (−0.42; 2.39)	0.169
Dyslipidemia (yes vs. no *)	0.29 (−0.44; 1.03)	0.434	0.12 (−1.13; 1.39)	0.842
BDS-22	−0.03 (−0.05; −0.01)	** 0.005 **	−0.03 (−0.08; 0.01)	0.144

* Reference group. Variables entered in the model: gender, age, marital status, work status, smoking status, CVD, hypertension, dyslipidemia, and BDS-22. Abbreviations: BDS-22: Beirut distress scale; CI: Confidence Interval; CVD: Cardiovascular diseases; LMDS: Lebanese Mediterranean Diet Score. ***p*-values in bold:** Statistically significant values.

**Table 5:** Multivariable analysis stratified according to dyslipidemia.

Linear Regression Analysis Taking the LMDS Scale as the Dependent Variable				
	No Dyslipidemia Subgroup		Dyslipidemia Subgroup	
	UB (95% CI)	*p*-Value	UB (95% CI)	*p*-Value
Gender (Female vs. Male *)	1.03 (0.49; 1.57)	** <0.001 **	0.40 (−0.81; 1.61)	0.517
Age	0.05 (0.03; 0.07)	** <0.001 **	0.04 (0.002; 0.08)	** 0.040 **
Marital status (Married vs. single *)	0.02 (−0.53; 0.57)	0.945	2.53 (1.30; 3.77)	** <0.001 **
Smoking (yes vs. No *)	−0.84 (−1.38; −0.29)	** 0.002 **	−0.38 (−1.54; 0.76)	0.508
Smoking (previous vs. No *)	−1.16 (−2.19; −0.12)	** 0.028 **	−0.59 (−2.34; 1.14)	0.501
Regular physical activity (yes vs. no *)	1.50 (0.97; 2.04)	** <0.001 **	1.21 (0.12; 2.30)	** 0.029 **
Work status (Employed vs. unemployed *)	0.14 (−0.41; 0.69)	0.615	1.03 (−0.26; 2.32)	0.119
CVD (yes vs. no *)	1.73 (1.05; 2.40)	** <0.001 **	0.14 (−0.92; 1.20)	0.793
Hypertension (yes vs. no *)	−0.27 (−0.92; 0.38)	0.416	−0.25 (−1.42; 0.90)	0.663
Diabetes (yes vs. no *)	0.72 (−0.10; 1.53)	0.087	0.51 (−0.60; 1.63)	0.370
BDS-22	−0.04 (−0.07; −0.02)	** <0.001 **	0.02 (−0.02; 0.06)	0.439

* Reference group. Variables entered in the model: gender, age, marital status, work status, smoking status, CVD, hypertension, dyslipidemia, and BDS-22. Abbreviations: BDS-22: Beirut distress scale; CI: Confidence Interval; CVD: Cardiovascular diseases; LMDS: Lebanese Mediterranean Diet Score. ***p*-values in bold:** Statistically significant values.

**Table 6:** Multivariable analysis stratified according to CVD.

Linear Regression Analysis Taking the LMDS Scale as the Dependent Variable				
	No CVD Subgroup		CVD Subgroup	
	UB (95% CI)	*p*-Value	UB (95% CI)	*p*-Value
Gender (Female vs. Male *)	1.21 (0.66; 1.77)	** <0.001 **	−0.77 (−1.82; 0.28)	0.152
Age	0.06 (0.04; 0.07)	** <0.001 **	−0.01 (−0.04; 0.02)	0.619
Marital status (Married vs. single *)	0.49 (−0.05; 1.04)	0.079	0.36 (−0.79; 1.53)	0.532
Smoking (yes vs. No *)	−0.76 (−1.30; −0.22)	** 0.005 **	−0.83 (−2.02; 0.34)	0.166
Smoking (previous vs. No *)	−0.89 (−1.95; 0.16)	0.096	−0.66 (−2.31; 0.98)	0.429
Regular physical activity (yes vs. no *)	1.51 (0.97; 2.05)	** <0.001 **	0.70 (−0.38; 1.78)	0.206
Work status (Employed vs. unemployed *)	0.41 (−0.15; 0.98)	0.154	0.02 (−1.14; 1.18)	0.973
Hypertension (yes vs. no *)	−0.44 (−1.08; 0.19)	0.174	0.34 (−0.89; 1.57)	0.587
Diabetes (yes vs. no *)	0.44 (−0.32; 1.22)	0.257	1.49 (0.23; 2.75)	** 0.021 **
Dyslipidemia (yes vs. no *)	0.64 (−0.12; 1.40)	0.100	−0.38 (−1.51; 0.74)	0.504
BDS-22	−0.02 (−0.04; 0.001)	** 0.047 **	−0.05 (−0.09; −0.01)	** 0.007 **

* Reference group. Variables in the model: gender, age, marital status, work status, smoking status, CVD, hypertension, dyslipidemia, and BDS-22. Abbreviations: BDS-22: Beirut distress scale; CI: Confidence Interval; CVD: Cardiovascular diseases; LMDS: Lebanese Mediterranean Diet Score. ***p*-values in bold:** Statistically significant values.

## Data Availability

The datasets used and/or analyzed during the current study are available from the corresponding author upon reasonable request due to ongoing research and planned follow-up studies.

## Supplementary Materials

The following supporting information can be downloaded at: https://www.mdpi.com/article/10.3390/nu16152426/s1, Table S1: LMDS Food Elements Adherence Mean.

## Abbreviations

ANOVA	Analysis of variance
BDS-22	Beirut Distress Scale
BMI	Body mass index
CAS	Central Administration of Statistics
CDC	Center for Disease Control and Prevention
CHD	Coronary heart disease
CI	Confidence Interval
COVID-19	Coronavirus disease 2019
CV	Cardiovascular
CVD	Cardiovascular disease
DALYs	Disability-adjusted life years
LMDS	Lebanese Mediterranean Diet Score
MedDiet	Mediterranean Diet
MI	Myocardial infarction
MUFA	Monounsaturated fatty acids
NCD	Non-Communicable Diseases
PUFA	Polyunsaturated fatty acids
RCBG	Random capillary blood glucose
SD	Standard deviation
SDG	Sustainable Development Goal
SPSS	Statistical Package for the Social Sciences
T2DM	Type 2 diabetes mellitus
UN	United Nations
UNESCO	United Nations Educational, Scientific and Cultural Organization
U.S.	United States
USD	United States Dollar
WC	Waist circumference
WHO	World Health Organization
