# Supplementary material for: Adherence to the Mediterranean Diet and Cardiovascular Risk Factors among the Lebanese Population: A Nationwide Cross-Sectional Post Hoc Study
Source: Nutrients. 2024 Jul 26;16(15):2426. doi: 10.3390/nu16152426 (PMC11313688; doi:10.3390/nu16152426)
Supplement: Supplementary file 1 [file nutrients-16-02426-s001.zip › nutrients-3072679-supplementary.pdf]

Supplementary File

Supplementary Table S1: LMDS Food Elements Adherence Mean

LMDS Food Elements	Mean ± SD
Raw Vegetables	2.10 ± 0.91
Stew	1.42 ± 0.78
Olive Oil	2.70 ± 0.97
Fish	1.18 ± 0.64
Grains	1.50 ± 0.75
White bread	2.39 ± 1.50
Whole Grain Bread	1.22 ± 1.47
Rice Pasta	1.55 ± 0.79
Cooked Vegetable	1.39 ± 0.80
Fruits	2.41 ± 1.00
Skimmed Milk	0.58 ± 0.98
Fast Food	2.96 ± 0.83
Fried Food	2.47 ± 0.94
Meat	2.23 ± 0.80
Sweets	2.34 ± 1.01
Full Fat Milk	2.53 ± 1.23

Abbreviations: LMDS: Lebanese Mediterranean Diet Score, SD: Standard Deviation
